# Performance of different spatial repellents (spatial emanators) against vector mosquito species in Mali, West Africa: a field trial using a non-human test method

**DOI:** 10.3389/finsc.2026.1811511

**Published:** 2026-04-21

**Authors:** Edita E. Revay, Karen McKenzie, Amy Junnila, Kristine Styer, Alexey M. Prozorov, Mohamed M. Traore, Liwang Cui, Roman V. Yakovlev, Aidas Saldaitis, Sekou F. Traore, Abdoul Habib Beavogui, Tatiana A. Prozorova, Gergely Petrányi, Ursula Benz, Rui-De Xue, Günter C. Müller

**Affiliations:** 1 Malaria Research and Training Center, Faculty of Medicine, Pharmacy and Odonto-Stomatology, University of Sciences, Techniques and Technology of Bamako, Bamako, Mali; 2 McKenzie Consulting and Research, LLC, Melbourne, FL, United States; 3 Paleo-DNA Laboratory, Department of Anthropology, Lakehead University, Thunder Bay, ON, Canada; 4 Woodstream Corp., Lancaster, PA, United States; 5 Faculty of Biology, Ludwig Maximilian University of Munich, Planegg-Martinsried, Germany; 6 Division of Infectious Diseases and International Medicine, Department of Internal Medicine, Morsani College of Medicine, University of South Florida, Tampa, FL, United States; 7 X-BIO Institute, University of Tyumen, Tyumen, Russia; 8 Institute of Biology, Ecology, Soil Science, Agriculture and Forestry, Tomsk State University, Tomsk, Russia; 9 Nature Research Centre, Vilnius, Lithuania; 10 Centre National de Formation et de Recherche en Santé Rurale de Maferinyah, Forécariah, Guinea; 11 Department of Medical Sciences, Gamal Abdel Nasser University, Conakry, Guinea; 12 Institute of Molecular Biology and Biotechnology, Foundation for Research and Technology-Hellas, Heraklion, Greece; 13 Zephyr Blue Foundation (Fóti Boglárka Alapítvány), Budapest, Hungary; 14 Division of Infectious Diseases and Tropical Medicine, Ludwig Maximilian University Hospital, Munich, Germany; 15 Anastasia Mosquito Control District, St. Augustine, FL, United States

**Keywords:** field efficacy, pyrethroids, spatial repellents, vector mosquitoes, West Africa

## Abstract

**Introduction:**

Spatial repellents (also called spatial emanators) are widely marketed for personal protection against mosquito bites, yet their real‑world performance varies substantially and is rarely evaluated under standardized field conditions. This study quantified the protective efficacy of six consumer‑available repellent devices at paired urban (*Aedes*-dominated) and rural (*Anopheles*-dominated) field sites in Mali, West Africa.

**Methods:**

Products included a Dynatrap® Mosquito Repellent electronic device with an 8.83% transfluthrin-containing replaceable heat-activated cartridge, a Thermacell^®^ E90 Rechargeable Mosquito Repeller electronic device with a 5.5% transfluthrin-containing replaceable heat-activated cartridge, two mosquito coils (Hassana containing 0.08% meperfluthrin and PIC^®^ containing 0.6% pyrethrins), a Cutter^®^ CitroGuard^®^ Candle containing 3% citronella oil, and an Isotronic mosquito repellent device with an oscillating frequency technology. Each product was tested in wind‑controlled V‑shaped field plots using three CO_2_‑baited CDC‑UV traps, positioned 14 ft from the device, to quantify spatial protection.

**Results:**

Across both sites, the heat-activated volatile pyrethroid devices achieved the strongest and most consistent reductions in mosquito captures. At the urban site, the Dynatrap^®^ and Thermacell devices lowered mean *Aedes* counts from all traps in all replicates 14.33 ± 2.05 in the control to 0.96 ± 0.27 and 0.88 ± 0.26, respectively, and reduced mean *Culex* counts from 8.83 ± 1.40 to 0.50 ± 0.21 and 1.00 ± 0.29, respectively. At the rural site, where *Anopheles* were predominant, these same devices decreased mean captures from 34.96 ± 7.20 in the control to 1.63 ± 0.49 (Dynatrap^®^) and 2.50 ± 0.63 (Thermacell^®^). *Culex* were reduced from 15.79 ± 2.91 to 2.63 ± 0.61 (Dynatrap^®^) and 4.00 ±0.69 (Thermacell^®^) at the same site. Dunnett’s multiple comparisons confirmed that these were the only treatments to produce significant reductions across multiple genera at both sites. Mosquito coils offered moderate but inconsistent protection, while the citronella candle and ultrasonic device showed minimal or no measurable effect, aligning with previous findings that citronella‑based products provide little field efficacy. This characterization of “inconsistent” reflects the night‑to‑night fluctuations in percent reduction, which are evident in the raw data (not shown) and already conveyed in the reported means and confidence intervals.

**Discussion:**

Together, these results demonstrate that heat‑activated volatile pyrethroid devices can deliver strong, broad‑spectrum spatial protection across ecologically varied environments. The study emphasizes the need for standardized, wind‑controlled field testing and highlights significant performance gaps among widely marketed consumer repellents. Given that spatial repellents are now formally recognized as a recommended intervention class in the 2025 WHO Guidelines, these performance gaps carry important public health implications, as inconsistent or underperforming products could undermine the protective value expected from this intervention category.

## 1 Introduction

Mosquito-borne diseases remain among the most significant public health challenges worldwide, with *Aedes*, *Culex*, and *Anopheles* mosquitoes transmitting pathogens responsible for malaria, dengue, chikungunya, Zika virus, West Nile virus, and other illnesses. In many regions, particularly across sub-Saharan Africa, vector control continues to rely heavily on long-lasting insecticidal nets (LLINs) and indoor residual spraying (IRS). While these interventions have substantially reduced malaria transmission, they provide limited protection outdoors or in peri-domestic spaces where human–mosquito contact frequently occurs. As a result, there is growing interest in spatial repellents, also referred to as spatial emanators, which can reduce mosquito landings in open or semi-open environments and complement existing vector control tools (1, 2). The 2025 WHO Guidelines for Malaria now formally recognize spatial repellents (emanators) as a recommended intervention class, noting that “effective protection of the population with spatial repellents depends on continuous high coverage in the target area, with duration of efficacy of the product and distribution model being the key determinants of feasibility and cost-effectiveness” (3). This updated guidance underscores the increasing relevance of spatial emanators within integrated vector management frameworks and highlights the need for rigorous, field-validated evaluations such as the present study.

Spatial repellents (or emanators) work by releasing volatile active ingredients that interfere with mosquito host-seeking behavior, decrease landings, or form protective zones around users. Volatile pyrethroids such as transfluthrin, metofluthrin, and allethrin have demonstrated strong potential in both laboratory and semi-field tests because of their quick action and ability to disperse effectively in the air (2). Nonetheless, the consumer market also offers a wide range of products—such as citronella candles, ultrasonic devices, and mosquito coils, that differ greatly in their active ingredients, delivery methods, and supporting evidence. Many of these products are heavily marketed despite having limited or inconsistent support from field validation (4, 5).

Citronella-based products remain popular due to their “natural” branding, yet numerous studies have shown that their protective effectiveness is minimal under real-world conditions. Müller and colleagues (6) found that citronella candles did not significantly reduce mosquito landings, concluding that their performance was indistinguishable from untreated controls. Ultrasonic devices have also failed to demonstrate repellency in controlled tests, despite widespread commercial claims (7). Mosquito coils, while capable of reducing biting pressure, often provide inconsistent protection and may be affected by wind, combustion rate, and environmental factors.

Despite widespread availability, few studies have compared multiple consumer spatial repellents simultaneously under standardized field conditions, and even fewer have assessed their performance across ecologically diverse environments. Variations in species composition, such as *Aedes* dominance in urban areas versus *Anopheles* in rural locations, may affect product effectiveness, yet these dynamics remain poorly understood. Furthermore, most field evaluations depend on human landing catches, which pose ethical and logistical challenges. Non-human test systems provide a safer alternative but have seldom been used in comparative field trials.

To fill these gaps, this study assessed the performance of six popular spatial repellent devices at paired urban and rural sites in Mali, West Africa, utilizing a standardized, wind-controlled, non-human testing method. The products included two heat-activated volatile pyrethroid devices (transfluthrin and allethrin/metofluthrin), two mosquito coils (meperfluthrin and pyrethrins), a citronella candle, and an ultrasonic plug-in device. The six products were selected to capture the diversity of consumer spatial repellents currently available on the global market, including heat-activated pyrethroid devices, passive emanators, botanical formulations, and portable devices. This ensured representation across active ingredient classes, delivery mechanisms, and product categories commonly used for outdoor mosquito protection.

Field trials were conducted in Mali due to its high malaria burden and the availability of ecologically distinct sites with abundant urban *Aedes/Culex* and rural *Anopheles* populations. The USTTB field stations maintain year-round mosquito activity and established infrastructure for standardized field evaluations, providing an ideal setting for reproducible testing across multiple mosquito genera.

To sharpen the scientific framing, we hypothesized that heat-activated volatile pyrethroid devices would provide significantly greater spatial protection than passive or non-pyrethroid consumer products, owing to their higher vapor pressure and more consistent release of active ingredients. We further hypothesized that relative product performance would remain consistent across the two ecologically distinct sites, despite differences in mosquito species composition, because the primary mode of action for volatile pyrethroids is expected to be broadly effective across *Aedes*, *Culex*, and *Anopheles* mosquitoes.

It should be noted that some spatial repellents can act through behavioral disruption of host-seeking (spatial repellency) or through knockdown and mortality at a distance (spatial killing), and heat-activated volatile pyrethroids may operate through either or both mechanisms. Because the CDC trap-based method (used here) measures reductions in mosquito captures surrounding repellent products, it captures the net decrease in host-seeking mosquitoes without distinguishing between these modes of action.

By measuring mosquito captures across the *Aedes*, *Culex*, and *Anopheles* genera and conducting rigorous statistical analyses, including Dunnett’s multiple-comparison tests, this study offers one of the most comprehensive evaluations to date of consumer spatial repellent performance under realistic field conditions.

## 2 Materials and methods

### 2.1 Study sites

Trial sites, one urban and one rural, were selected based on the presence of mosquitoes from the three genera *Anopheles*, *Culex*, and *Aedes*, as well as sufficient biting pressure and suitable microclimate (areas with little to no air movement naturally protected from wind) during December 2023 to January 2024. At the rural site, located on the floodplain of the River Niger and primarily hosting *Anopheles* and *Culex* mosquitoes, monitoring took place from 11:00 PM to 1:00 AM. Meanwhile, at the urban site in the suburban neighborhood of Sebenikoro on the outskirts of Bamako, where *Aedes* and *Culex* are predominant, monitoring was conducted from 8:00 to 10:00 PM. Both sites are known to the USTTB test facility for maintaining year-round high and stable biting pressure for all three mosquito genera: *Aedes*, *Culex*, and *Anopheles*.

Field trials were conducted at two ecologically distinct locations in Mali, West Africa: an urban site characterized by high densities of *Aedes* and *Culex* mosquitoes, and a rural site dominated by *Anopheles* species. Pre-trial surveillance using CO_2_-enhanced CDC light traps confirmed the presence and relative abundance of *Aedes*, *Culex*, and *Anopheles* at both sites. Trials were performed during the peak evening biting period under low-wind conditions (0–4 km/h) to ensure stable plume formation and minimize environmental variability.

The study was conducted during the December–January dry season because baseline entomological assessments confirmed that mosquito densities met the minimum thresholds required for reliable product evaluation (see 2.6 Pre-trial Assessment), and environmental conditions were more stable than in the wet season (see 2.3 Plot Design). In this context, environmental stability and adequate catch density were prioritized over seasonal variation in species composition to ensure consistent and interpretable comparisons amongst products.

### 2.2 Repellent devices evaluated

**Figure 1:**
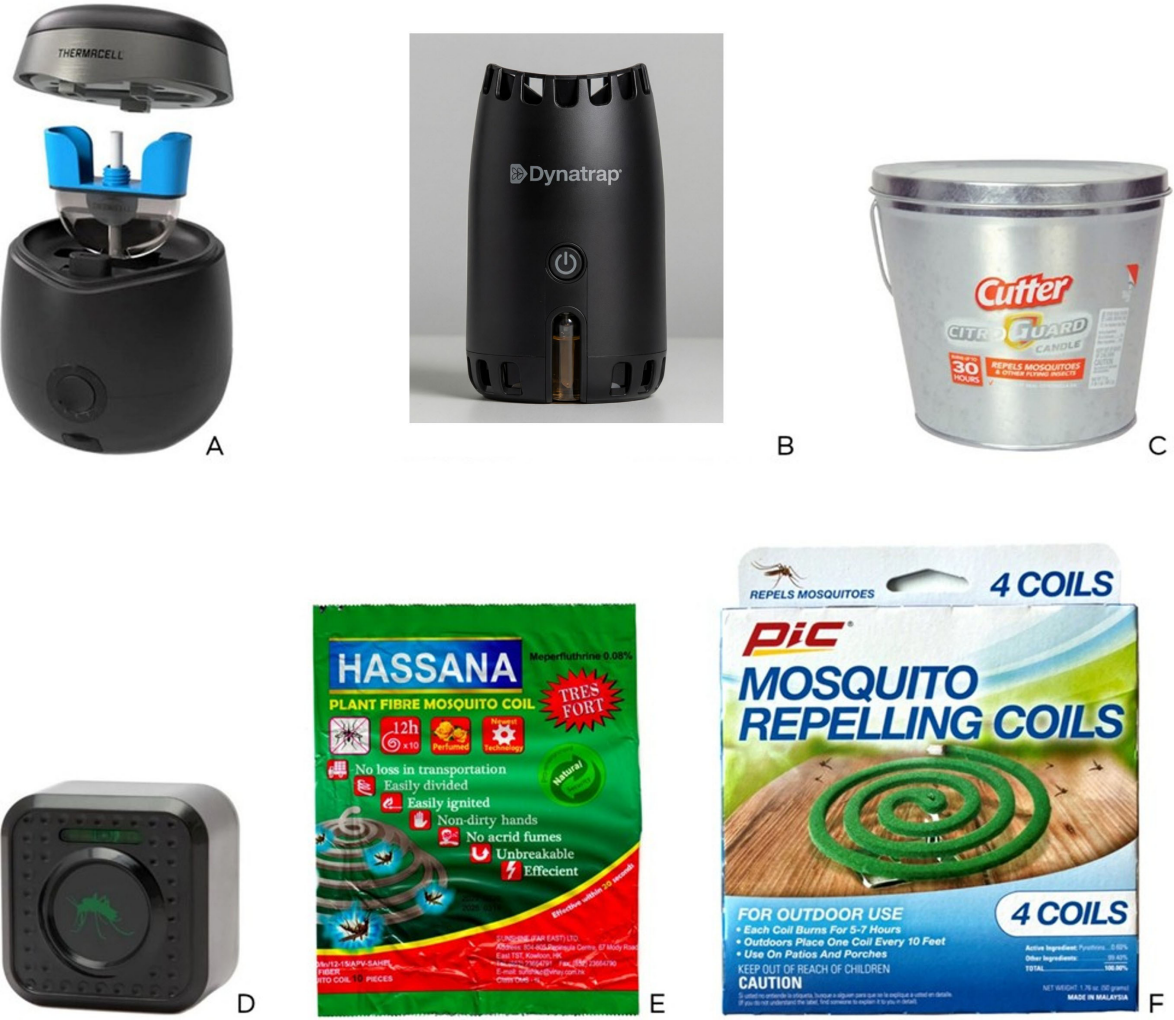
Six commercially available spatial repellents tested in the study. **(A)** Thermacell^®^ E90 Rechargeable Mosquito Repeller electronic device with a 5.5% transfluthrin-containing replaceable cartridge. **(B)** Dynatrap^®®^ Mosquito Repellent electronic device with an 8.83% transfluthrin-containing replaceable heat-activated cartridge. **(C)** Cutter^®^ CitroGuard^®^ Candle containing 3% citronella oil. **(D)** Isotronic^®^ mosquito repellent “eye” device with an oscillating frequency technology (ultrasonic). **(E)** Hassana mosquito coil containing 0.08% meperfluthrin. **(F)** PIC^®^ mosquito repelling coil containing 0.6% pyrethrins.

Six commercially available spatial repellent products (**Figure 1**) were selected to represent the major categories of consumer mosquito-control devices:

1. Thermacell^®^ E90 Rechargeable Mosquito Repeller electronic device with a 5.5% transfluthrin-containing replaceable cartridge (further called Thermacell or Therma, in tables).
2. Dynatrap^®^ Mosquito Repellent electronic device with an 8.83% transfluthrin-containing replaceable heat-activated cartridge (further called Dynatrap^®^).
3. Cutter^®^ CitroGuard^®^ Candle containing 3% citronella oil (further called Candle).
4. Isotronic^®^ mosquito repellent “eye” device with an oscillating frequency (ultrasonic) technology (further called Ultrasonic device or Ultra, in tables).
5. Hassana mosquito coil containing 0.08% meperfluthrin (further called Coil 1).
6. PIC^®^ Mosquito Repelling Coil containing 0.6% pyrethrins (further called Coil 2).

All products were tested following the manufacturer’s instructions. Devices requiring heat activation were powered by portable battery packs that supplied a stable current to maintain consistent operation during each trial. Full fresh coils were used for each replicate and burned for the entire length of the 2 hour trial period.

### 2.3 Experimental plot design

This study provides standardized, wind-controlled field evaluations of consumer spatial repellents across distinct mosquito communities in Mali, with all urban and rural trial plots precisely georeferenced using GPS coordinates to ensure reproducible site characterization. Rural site GPS coordinates are: Latitude: N12.112674° x Longitude: W08.320109° while the urban site coordinates are: Latitude: N12.565434° x Longitude: W08.068171°.

**Figure 2:**
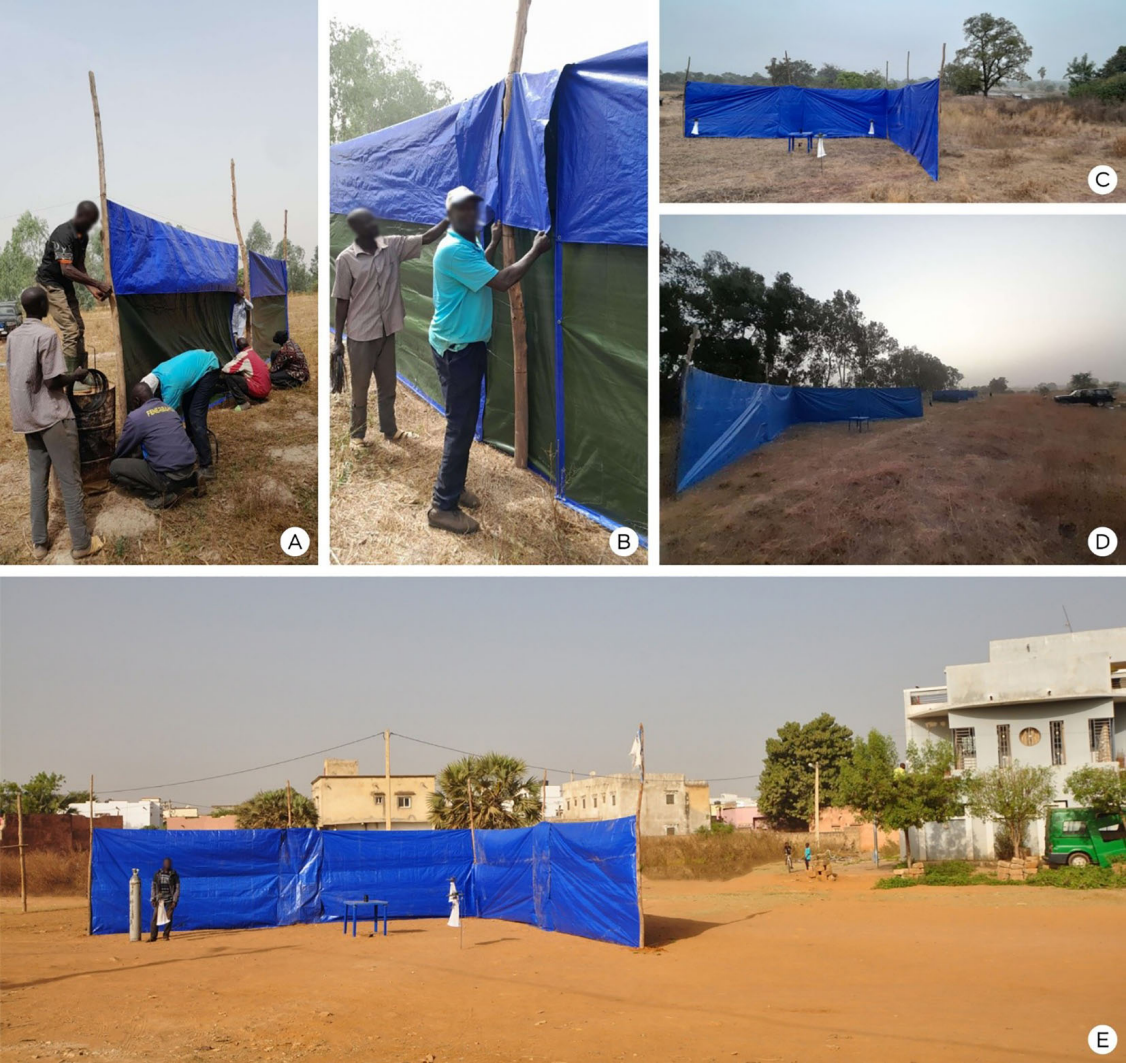
Field technicians erecting the 30 ft × 8 ft tarp walls forming the 90° V-shaped structure used to stabilize the repellent plume **(A, B)**. The V-shaped structure in the rural **(C, D)** and urban **(E)** sites.

Each test site was large enough to accommodate seven independent 20 ft × 20 ft test plots, with a minimum 50 ft buffer zone between plots to prevent plume overlap and cross-contamination (**Figure 2**). Each replicate consisted of one untreated control plot and one treatment plot for each of six products (N = 7 total), all operated simultaneously.

Because spatial repellent performance depends heavily on airflow, the initial wind direction at each site was determined from historical weather station data and confirmed during preliminary visits with smoke cartridges. Just before each trial, wind direction was re-verified with flagging tape placed at the plot centers. Testing proceeded only when wind speeds inside and immediately around the plots were 0–4 km/h (0–2.5 mph) to ensure stable plume formation.

Each plot included two heavy-duty polyethylene tarp walls (30 µm thick) set at a fixed 90° angle, with the exterior vertex facing directly into the wind. Each wall measured 30 ft in length and 8 ft in height, forming a semi-enclosed V-shaped structure designed to stabilize the repellent plume and minimize lateral dispersion. The repellent device was positioned at the geometric center of this structure on a 30-inch-high stand, ensuring consistent elevation across replicates.

To measure mosquito activity in the repellent field, three CO_2_-baited New Standard Miniature Black Light (UV) Traps (Model 1212, John Hock, Gainesville) were set up per plot. CO_2_ flow was maintained at 500 mL/min (± 3%) using calibrated flow regulators, and traps were turned on and off simultaneously with CO_2_ release. All traps were mounted 24 inches above the ground.

Trap placement followed a standardized geometric design based on the manufacturer’s 20 ft × 20 ft area-coverage claim. The diagonal (C) of this square was calculated as follows:


$$\text{The radius }\left(\text{R}\right)\text{ of the protection circle was}:{\text{ C}}^{2}={\text{ A}}^{2}+{\text{B}}^{2};\text{and then R }=\text{ C}/2$$

**Figure 3:**
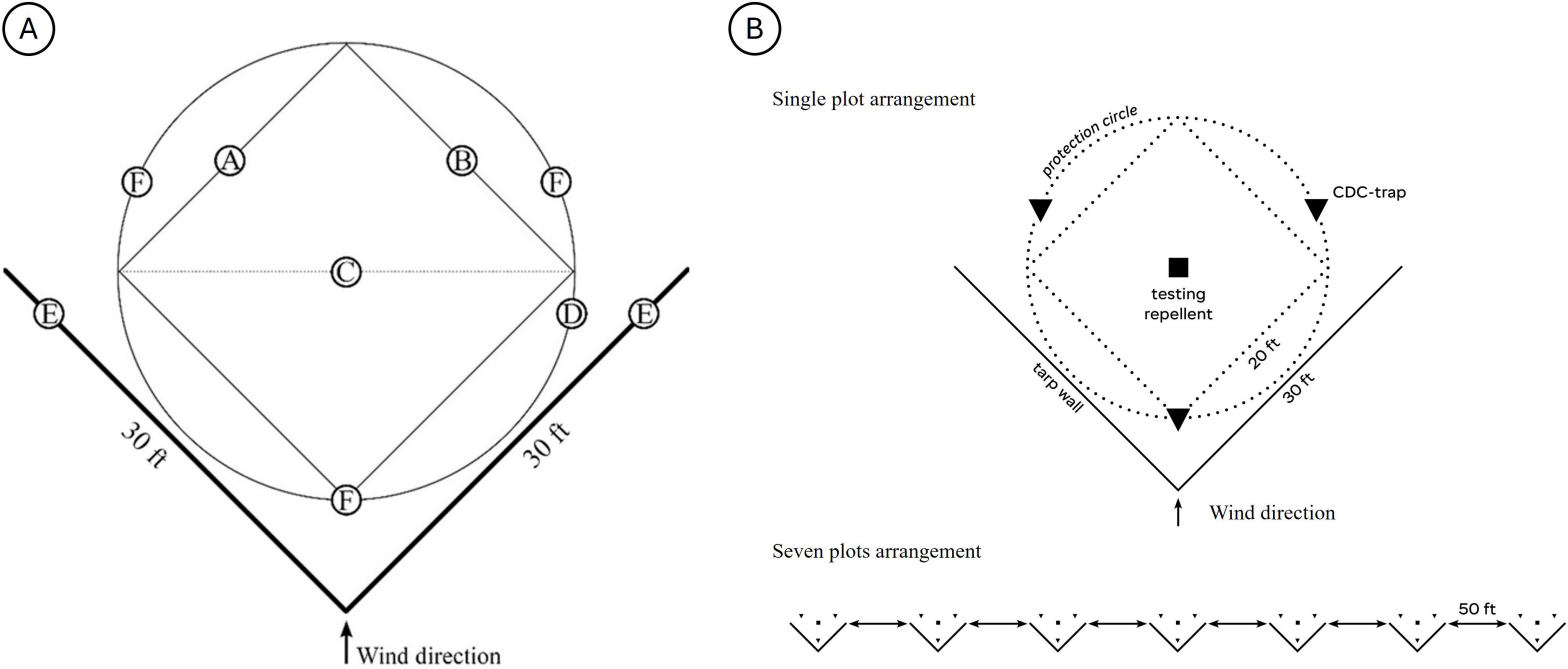
**(A)** Calculation of the protection area and CDC trap placement. **(A, B)** Sides of the 20 ft × 20 ft square defining the repellent claim area. **(C)** The diagonal of the manufacturer-specified 20 × 20 ft coverage square, which corresponds to the diameter of the circular protection zone used for trap placement-also with repellent product in the center. **(D)** Protection circle where CDC-UV traps were placed. **(E)** V-shaped tarp wall. **(F)** Placement of CDC traps. **(B)** Single plot arrangement (with triangles representing CDC traps and the square showing placement of the repellent) and the 7 plots, in a line, with 50ft between them.

This 14 ft radius defined the distance from the central device to each trap. Trap 1 was positioned 4 ft from the apex of the tarp walls on the inside of the structure. The other two traps were placed at 120° intervals around the center point, creating an equilateral triangle at equal distances from the repellent source. This layout ensured all traps sampled from the same protection boundary and experienced the same exposure to the repellent plume (**Figure 3**).

One product was tested per plot each night, and the control plot used the same setup without a repellent device. This standardized, wind-stabilized design ensured consistent plume formation, reproducible trap exposure, and straightforward comparison of mosquito activity across treatments, nights, and sites.

Environmental conditions, including temperature, humidity, and wind speed, were recorded throughout each trial. Nights with wind speeds over 4 km/h were excluded to ensure plume stability.

### 2.4 Trial procedures

Each trial began at dusk and ran for the standardized collection period of 2 hours (8:00 to 10:00 pm at the urban site and 11:00 pm to 1:00 am at the rural site). CO_2_ was supplied to each trap using CO_2_ canisters ensure consistent release rates. Trap catches were collected at the end of each trial, and mosquitoes were transported to the laboratory for identification.

Mosquitoes were identified to genus (*Aedes*, *Culex*, *Anopheles*) and species using morphological keys under a stereomicroscope. Counts from the three traps within each plot were pooled to generate a nightly mean for each treatment. Each treatment was replicated eight times per site (n = 8). For every replicate, mosquito counts were recorded separately for each genus. This produced a balanced dataset suitable for parametric analysis. To minimize any areal or locational bias, the six products were systematically rotated through the plots across replicates so that each treatment occupied each plot position an equal number of times.

### 2.5 Statistical analysis

The number of mosquitoes collected per trap was recorded, and totals were calculated for each plot. Averages ± standard deviations (SD) and standard errors (SE) were computed for all treatment and control conditions using the equation average = Σ#mosquitoes collected/# of traps. Standard error was calculated by: Standard error = Standard deviation/√N, where N is the number of replicates.

Shapiro–Wilk tests were conducted on all datasets from both sites to evaluate normality. Based on post-test results, a two-way ANOVA was performed for the comparative experiment, in which all products were tested, with separate analyses for the urban and rural sites with product and mosquito species as fixed factors. Nightly replicates contributed to the residual variance and were therefore treated as repeated measurements rather than as an independent factor. To contextualize the magnitude of treatment effects, η² effect sizes were calculated from the ANOVA sums of squares. At both sites, product identity explained the largest proportion of variance, followed by species differences and a moderate product × species interaction.

When the ANOVA indicated significant main effects or interactions, Dunnett’s multiple comparisons test was used to compare each product directly with the untreated control. Dunnett’s test was selected because it controls the family-wise error rate while focusing specifically on comparisons of interest (each product vs. control). For each comparison, the mean difference, 95% confidence interval, q-value, degrees of freedom, and p-value were reported.

### 2.6 Pre-trial assessment

To confirm that each trial site had adequate mosquito fauna composition and sufficient biting pressure before initiating the efficacy trials, baseline mosquito collections were conducted over two consecutive nights (November 29–30, 2023) at both the urban and rural locations. Ten CO_2_-enhanced CDC-UV traps were operated for two hours during the peak activity period of the local mosquito populations determined by the field scientists, based on prior experience and by monitoring the mosquito activity on an hourly basis, from 8:00 pm to 1:00 am, at both sites. A minimum threshold of one mosquito per minute per plot) equivalent to 60 mosquitoes per plot per hour) was required for a site to qualify for inclusion in the study. Pretreatment monitoring results confirming that both sites met the required mosquito pressure are presented in **Table 1**.

**Table 1:** Mosquito species composition of the pre-treatment phase at the two sites.

Species/	Rural site				Urban site			
species complex (s.l.)	Number	% of	Genus	Genus	Number	% of	Genus	Genus
	specimens	sample	total	%	specimens	sample	total	%
*Culex pipiens* s.l.	135	33.75%			103	25.75%		
*Culex neavei*	9	2.25%			4	1.00%		
*Culex perexiguus*	2	0.50%	146	36.5%	36	9.00%	143	35.75%
*Aedes aegypti*	0	0.00%			145	36.25%		
*Aedes albopictus*	0	0.00%			73	18.25%		
*Aaedes sudanensis*	0	0.00%	0	0.00%	4	1.00%	222	55.50%
*Anopheles gambiae* s.l.	243	60.75%			35	8.75%		
*Anopheles funestus*	0	0.00%			0	0.00%		
*Anopheles rufipes*	8	2.00%	251	62.75%	0	0.00%	35	8.75%
*Uranotaenia* sp.	3	0.75%	3	0.75%	0	0.00%	0	0.00%
TOTAL	400	100.00%	400	100.00%	400	100.00%	400	100.00%

Pretreatment monitoring confirmed that both sites exceeded this threshold (**Table 1**). From these baseline collections, a random subsample of 400 female mosquitoes per site (40 individuals from each of the 10 traps) was selected for taxonomic identification. Female mosquitoes were sorted and identified to genus using standard morphological keys under a stereomicroscope.

## 3 Results

Baseline collections were performed over two consecutive nights, with ten CO_2_-enhanced CDC-UV traps that were set up for two hours at both the urban and rural locations during the peak activity times of the local mosquito populations. A minimum threshold of one mosquito per minute per plot (equivalent to 60 mosquitoes per plot per hour) was necessary for a site to qualify for inclusion in the study. Pretreatment monitoring showed that both sites exceeded this threshold (**Table 1**).

From these baseline collections, a random subsample of 400 female mosquitoes per site (ten individuals from each of ten traps) was chosen for taxonomic identification. Female mosquitoes were sorted and identified to genus using standard morphological keys under a stereomicroscope. The resulting species-composition profiles for each site are shown in **Table 1**.

The minimum mosquito pressure required to qualify the test site for inclusion in this study was one mosquito collected per minute per plot (e.g., a treatment with one-hour monitoring requires 60 mosquitoes collected per plot per hour). For pretreatment monitoring results verifying the needed mosquito pressure, see **Table 1**.

### 3.1 Urban site results

#### 3.1.1 Normality assessment

All mosquito count datasets from the urban site passed the Shapiro–Wilk normality test (α = 0.05), supporting the use of parametric analyses (**Table 2**).

**Table 2:** Shapiro–Wilk normality test results for mosquito count data from the urban field site.

Urban data							
Shapiro–Wilk test	Control	Candle	Ultra	Coil 1	Coil 2	Dynatrap^®^	Thermacell
W	0.9988	0.9842	0.75	0.8787	0.8663	0.9962	0.8929
P value	0.9333	0.7592	0.529	0.3207	0.285	0.8819	0.3631
Passed (alpha=0.05)?	Yes	Yes	Yes	Yes	Yes	Yes	Yes

All mosquito count datasets passed the Shapiro–Wilk normality test (α = 0.05), indicating that parametric analyses were appropriate. Dunnett’s multiple comparisons test revealed clear differences in product performance. For *Aedes*, both heat-activated volatile pyrethroid devices—Dynatrap and Thermacell—produced large, statistically significant reductions relative to the control (mean differences of 13.37 and 13.46, respectively; p < 0.0001). The two mosquito Coils and the ultrasonic device also significantly reduced *Aedes* captures, though with smaller effect sizes and greater variability. The Candle did not differ significantly from the control.

#### 3.1.2 Summary of raw trap-count results (urban site—all genera, all products)

**Figure 4:**
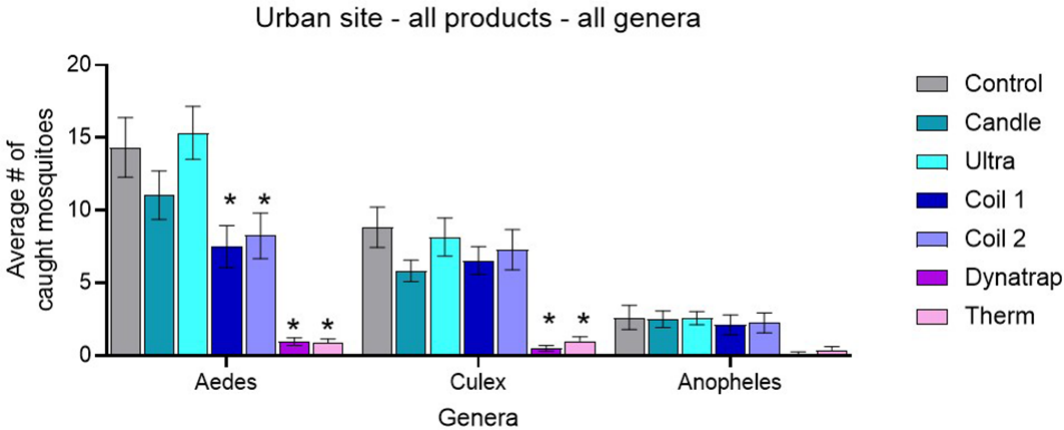
Mean mosquito captures ± SEM for all products across all genera at the urban field site. * indicates significant difference against control.

Across all three mosquito genera (*Aedes*, *Culex*, and *Anopheles*), the untreated control plots consistently produced the highest mean trap counts, confirming strong and stable mosquito pressure during the urban-site trials (**Figure 4**).

For *Aedes*, the control mean was 14.33 ± 2.05 (n = 8), substantially higher than the means for some treatment conditions. The Candle (11.04 ± 1.67) and Ultrasonic device (15.33 ± 1.83) showed no real consistent reductions, while Coil 1 (7.50 ± 1.44) and Coil 2 (8.25 ± 1.57) produced moderate decreases. The two heat-activated volatile pyrethroid devices, Dynatrap^®^ (0.96 ± 0.27) and the Thermacell (0.88 ± 0.26), yielded the lowest *Aedes* counts, indicating the strongest suppression.

A similar pattern was observed for *Culex*. The control mean was 8.83 ± 1.40, with the Candle (5.83 ± 0.74), Ultrasonic device (8.17 ± 1.30), Coil 1 (6.54 ± 0.96), and Coil 2 (7.29 ± 1.38) showing limited or inconsistent reductions. In contrast, Dynatrap^®^ (0.50 ± 0.21) and the Thermacell (1.00 ± 0.29) again produced the lowest counts, demonstrating the most effective reduction in *Culex* activity.

For *Anopheles*, overall captures were low across all treatments, reflecting the naturally low abundance of this genus at the urban site. The control mean was 2.63 ± 0.84, with treatment means ranging from 2.50 ± 0.58 (Candle) to 0.13 ± 0.12 (Dynatrap^®^). Although the heat-activated volatile pyrethroid devices produced the lowest numerical counts, none of the differences reached statistical significance due to low baseline numbers.

These raw trap-count patterns align with the statistical outcomes: heat-activated volatile pyrethroid devices consistently produced the greatest reductions across all genera, Coils provided moderate reductions, and the Candle and Ultrasonic device showed minimal or no measurable effect.

#### 3.1.3 Two-way ANOVA and Dunnett’s multiple comparisons

Following a significant ANOVA score (p < 0.05; see **Supplementary Table S1**), a Dunnett’s post-hoc comparisons (Post two-way ANOVA) were used to determine which treatments differed significantly from the untreated control for each mosquito genus. The results revealed strong genus-specific patterns in product performance, with clear distinctions between ineffective, moderately effective, and highly effective treatments (**Table 3**).

**Table 3:** Dunnett’s multiple comparisons test showing mean differences, confidence intervals, values, degrees of freedom, and significance for all treatments relative to the control at the urban field site.

Dunnett’s multiple comparisons test						
Urban site	Mean diff	95.00% CI of diff	Significant?	q	DF	P value
*Aedes*						
Control vs. Candle	3.292	-0.4087 to 6.992	No	2.31	147	0.1007
Control vs. Ultra	-1.000	-4.960 to 2.960	No	0.6559	147	0.9683
Control vs. Coil 1	6.833	3.133 to 10.53	Yes	4.796	147	<0.0001
Control vs. Coil 2	6.083	2.383 to 9.784	Yes	4.27	147	0.0002
Control vs. Dynatrap^®^	13.37	9.675 to 17.08	Yes	9.387	147	<0.0001
Control vs. Therm	13.46	9.758 to 17.16	Yes	9.446	147	<0.0001
*Culex*						
Control vs. Candle	3	-0.7004 to 6.700	No	2.106	147	0.1582
Control vs. Ultra	0.6667	-3.034 to 4.367	No	0.4679	147	0.9941
Control vs. Coil 1	2.292	-1.409 to 5.992	No	1.608	147	0.3971
Control vs. Coil 2	1.542	-2.159 to 5.242	No	1.082	147	0.7682
Control vs. Dynatrap^®^	8.333	4.633 to 12.03	Yes	5.849	147	<0.0001
Control vs. Therm	7.833	4.133 to 11.53	Yes	5.498	147	<0.0001
*Anopheles*						
Control vs. Candle	0.125	-3.575 to 3.825	No	0.08773	147	>0.9999
Control vs. Ultra	0.04167	-3.659 to 3.742	No	0.02924	147	>0.9999
Control vs. Coil 1	0.5	-3.200 to 4.200	No	0.3509	147	0.9988
Control vs. Coil 2	0.375	-3.325 to 4.075	No	0.2632	147	0.9998
Control vs. Dynatrap^®^	2.495	-1.206 to 6.195	No	1.751	147	0.3132
Control vs. Therm	2.25	-1.450 to 5.950	No	1.579	147	0.4157

q value—accounts for the number of groups being compared to the control; the greater the value, the more likely significant is the difference between the item and the control.

DF, degrees of freedom.

Across all genera, the Candle and Ultrasonic device rarely differed from the control, while the two Coils showed mixed performance. In contrast, the Dynatrap^®^ and Thermacell consistently produced the largest and most statistically robust reductions, particularly for *Aedes* and *Culex*.

For *Aedes*, the Candle did not differ significantly from the control (mean diff. = 3.29, 95% CI: –0.41 to 6.99, p = 0.1007). In contrast, all other treatments produced highly significant reductions. The Ultrasonic device showed no significant decrease (mean diff. = -1.00 p =0.9683), as did Coil 1 (mean diff. = 6.83, p < 0.0001) and Coil 2 (mean diff. = 6.08, p = 0.0002). The strongest effects were observed for the Dynatrap^®^ (mean diff. = 13.37, p < 0.0001) and the Thermacell (mean diff. = 13.46, p < 0.0001), both of which produced the highest q-values and the most pronounced reductions in *Aedes* captures.

For *Culex*, most products did not differ significantly from the control. The Candle, Ultrasonic device, and both Coils showed no significant reductions, with confidence intervals crossing zero and p-values ranging from 0.1582 to 0.9941. Only the Dynatrap^®^ and Thermacell produced statistically significant decreases in *Culex* captures. The Dynatrap^®^ showed a mean difference of 8.33 (95% CI: 4.63 to 12.03, p < 0.0001), and the Thermacell showed a similar effect (mean diff. = 7.83, 95% CI: 4.13 to 11.53, p < 0.0001). These two treatments were the only products to significantly outperform the control for this genus.

For *Anopheles*, none of the products differed significantly from the control. All comparisons yielded small mean differences with wide confidence intervals that crossed zero, and all p-values were non-significant (ranging from 0.3132 to >0.9999). Even the Dynatrap^®^ (mean diff. = 2.50) and Thermacell (mean diff. = 2.25) did not reach statistical significance for this genus, likely reflecting the low overall abundance of *Anopheles* at the urban site and the resulting reduced statistical power.

### 3.2 Rural site results

#### 3.2.1 Test for normality

All mosquito count datasets from the rural field site met the assumption of normality (**Table 4**). Shapiro–Wilk tests conducted for each treatment, control, Candle, Ultrasonic device, both Coils, and the two heat-activated volatile pyrethroid devices returned non-significant results (α = 0.05), indicating that none of the distributions deviated from normality. Test statistics (W = 0.9268–0.9978) and corresponding p-values (0.4767–0.9108) confirmed that all treatments passed the normality check. These results support the use of parametric analyses for evaluating treatment effects at the rural site.

**Table 4:** Shapiro–Wilk normality test results for mosquito count data from the rural field site.

Rural data							
Shapiro–Wilk test	Control	Candle	Ultra	Coil 1	Coil 2	Dynatrap^®^	Therm
W	0.9969	0.984	0.9978	0.9268	0.9922	0.9815	0.9796
P value	0.8937	0.758	0.9108	0.4767	0.8316	0.7391	0.7262
Passed (alpha=0.05)?	Yes	Yes	Yes	Yes	Yes	Yes	Yes

#### 3.2.2 Rural site—summary of raw trap−count results (all genera, all products)

**Figure 5:**
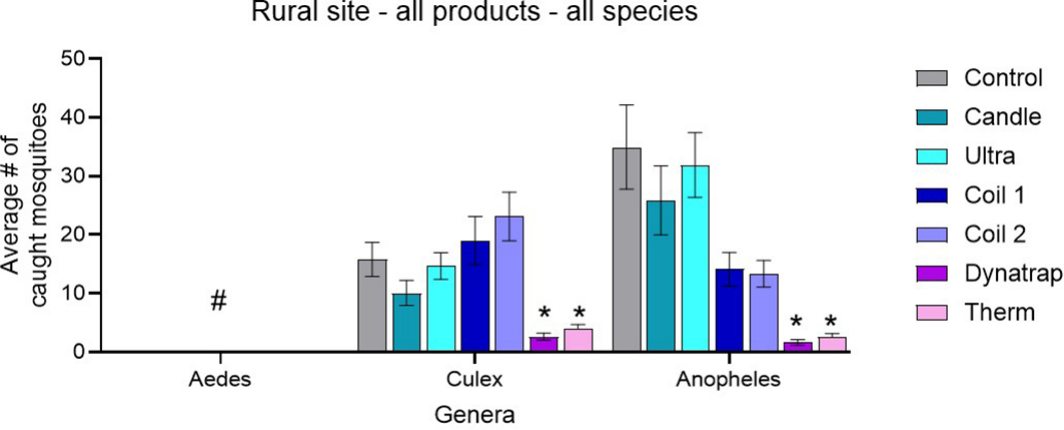
Mean ± SEM number of *Aedes*, *Culex*, and *Anopheles* mosquitoes captured per treatment during rural-site trials. ^#^No *Aedes* were caught at the rural site. *Indicates significant difference against control.

Across all mosquito genera, the untreated control plots at the rural site yielded the highest trap counts, confirming strong, consistent mosquito pressure during the trials (**Figure 5**).

For *Aedes*, all treatments, including the control, recorded 0.00 ± 0.00 (n = 8), reflecting the complete absence of this genus at the rural site during the sampling period. Because no *Aedes* were captured under any condition, no treatment-related differences could be evaluated.

For *Culex*, the control mean was 15.79 ± 2.91 (n = 8). The Candle (10.08 ± 2.16) and Ultrasonic device (14.67 ± 2.29) produced only partial or inconsistent reductions relative to the control. Coil 1 (19.00 ± 4.10) and Coil 2 (23.13 ± 4.14) yielded higher mean captures than the control, indicating no protective effect. In contrast, the heat-activated volatile pyrethroid devices, Dynatrap^®^ (2.63 ± 0.61) and Thermacell (4.00 ± 0.69), produced the lowest *Culex* counts, demonstrating the strongest suppression.

For *Anopheles*, the control mean was 34.96 ± 7.20, with the Candle (25.88 ± 5.89) and Ultrasonic device (31.92 ± 5.54) showing partial reductions but still substantial activity. Coil 1 (14.13 ± 2.87) and Coil 2 (13.33 ± 2.26) produced more pronounced decreases. As with *Culex*, the lowest *Anopheles* captures were observed for the heat-activated volatile pyrethroid devices: Dynatrap^®^ at 1.63 ± 0.49 and Thermacell at 2.50 ± 0.63, representing the strongest reductions among all products.

Overall, the rural-site trap-count data show a consistent pattern: Dynatrap^®^ and Thermacell produced the greatest reductions across genera, the Coils provided moderate suppression for *Anopheles* but not *Culex*, and the Candle and Ultrasonic device showed minimal or inconsistent effects. These patterns reflect the site’s species composition, where *Culex* and *Anopheles* were abundant and *Aedes* was absent during the trial period.

#### 3.2.3 Two-way ANOVA with Dunnett’s multiple comparisons

Following a significant ANOVA score (p < 0.005; see **Supplementary Table S2**), a Dunnett’s multiple comparisons test revealed distinct, genus-specific patterns in treatment performance at the rural field site (**Table 5**). For *Aedes*, no treatment differed significantly from the control. All mean differences were zero, confidence intervals were wide and centered on zero, and all p-values exceeded 0.9999. This uniform non-significance reflects the low and inconsistent *Aedes* activity at the rural site, which limited the ability to detect treatment effects.

**Table 5:** Dunnett’s multiple comparisons test showing mean differences, confidence intervals, values, degrees of freedom, and significance for all treatments relative to the control at the rural field site.

Dunnett’s multiple comparisons test						
Rural site	Mean diff.	95.00% CI of diff.	Significant?	q	DF	P value
*Aedes*						
Control vs. Candle	0	-10.88 to 10.88	No	0	147	>0.9999
Control vs. Ultra	0	-10.88 to 10.88	No	0	147	>0.9999
Control vs. Coil 1	0	-10.88 to 10.88	No	0	147	>0.9999
Control vs. Coil 2	0	-10.88 to 10.88	No	0	147	>0.9999
Control vs. Dynatrap^®^	0	-10.88 to 10.88	No	0	147	>0.9999
Control vs. Therm	0	-10.88 to 10.88	No	0	147	>0.9999
*Culex*						
Control vs. Candle	5.708	-5.176 to 16.59	No	1.362	147	0.5661
Control vs. Ultra	1.125	-9.759 to 12.01	No	0.2684	147	0.9997
Control vs. Coil 1	-3.208	-14.09 to 7.676	No	0.7656	147	0.9366
Control vs. Coil 2	-7.333	-18.22 to 3.551	No	1.75	147	0.3138
Control vs. Dynatrap^®^	13.17	2.283 to 24.05	Yes	3.142	147	0.0107
Control vs. Therm	11.79	0.9078 to 22.68	Yes	2.814	147	0.0280
*Anopheles*						
Control vs. Candle	9.083	-1.801 to 19.97	No	2.167	147	0.1386
Control vs. Ultra	3.042	-7.842 to 13.93	No	0.7258	147	0.9497
Control vs. Coil 1	20.83	9.949 to 31.72	Yes	4.971	147	<0.0001
Control vs. Coil 2	21.62	10.74 to 32.51	Yes	5.16	147	<0.0001
Control vs. Dynatrap^®^	33.33	22.45 to 44.22	Yes	7.954	147	<0.0001
Control vs. Therm	32.46	21.57 to 43.34	Yes	7.745	147	<0.0001

q value—accounts for the number of groups being compared to the control; the greater the value, the more likely significant is the difference between item and control.

DF, degrees of freedom.

For *Culex* in the rural environment, most treatments did not differ significantly from the control. The Candle, Ultrasonic device, and Coil 1 all showed non-significant differences, with confidence intervals overlapping zero. However, two treatments produced significant reductions in *Culex* captures: Dynatrap^®^ (mean difference 13.17; p = 0.0125), and Thermacell (mean difference 11.79; p = 0.0280). These results indicate that *Culex* responded most strongly to the heat-activated volatile pyrethroid devices and, unexpectedly, to Coil 2.

For *Anopheles*, several treatments produced significant reductions relative to the control. The Candle showed and the Ultrasonic device showed no significant decrease, but both Coils resulted in moderate decreases while Dynatrap^®^ and Thermacell resulted in substantial decreases in *Anopheles* captures, with mean differences of from 33.33 and 32.46 as well as p-values < 0.0001. These strong effects reflect the high baseline abundance of *Anopheles* at the rural site, which provided sufficient statistical power to detect treatment differences.

Overall, the rural-site results demonstrate that treatment performance was strongly genus-dependent. No product significantly reduced *Aedes*, while *Culex* showed selective responsiveness, primarily to heat-activated volatile pyrethroid devices. In contrast, *Anopheles* exhibited broad and pronounced reductions across multiple treatments. These findings highlight the ecological specificity of repellent performance and underscore the importance of local species composition when evaluating spatial repellent efficacy.

## 4 Discussion

Across both urban and rural environments, this study demonstrates a consistent and robust hierarchy in the performance of commercially available spatial repellents. Despite substantial differences in species composition between sites—*Aedes* dominating the urban environment and *Anopheles* dominating the rural environment—product efficacy followed the same overarching pattern. Heat-activated volatile pyrethroid devices, Dynatrap^®^ and Thermacell^®^, consistently produced the lowest mosquito captures and the strongest reductions relative to untreated controls. These findings align with extensive prior work showing that volatilized pyrethroids such as transfluthrin and metofluthrin create effective protective zones, disrupt host-seeking behavior, and reduce mosquito approach and landing rates across multiple genera (1, 8–11). Reviews of airborne pyrethroids similarly highlight their ability to interfere with orientation and olfactory-guided host detection, supporting the strong performance observed here (2, 12).

In contrast, the tested citronella candle and the ultrasonic device provided little to no measurable protection at either site. Their performance was statistically indistinguishable from the control in nearly all comparisons. These results reinforce long-standing evidence that citronella-based products offer minimal or short-lived repellency under field conditions (4, 13–15). Multiple evaluations have shown that citronella candles fail to reduce mosquito landings or biting pressure in realistic outdoor settings (6, 7). Similarly, ultrasonic devices have repeatedly been shown to provide no protective effect and are not supported by any mechanistic evidence of mosquito behavioral disruption (15–17). The present study corroborates these findings across two ecologically distinct environments, underscoring the limited utility of citronella candles and ultrasonic devices as primary mosquito-avoidance strategies.

Mosquito coils produced moderate reductions in some contexts, particularly for *Aedes* in the urban environment and *Anopheles* in the rural environment, but their effects were inconsistent and substantially weaker than those of the heat-activated volatile pyrethroid devices. This pattern is consistent with prior evaluations showing that coils can reduce biting pressure but often fail to provide uniform or reliable protection, with performance varying by species, airflow, and formulation (18–20). At the rural site, the PIC^®^ coil even produced slightly higher *Culex* captures than the control, suggesting behavioral interference or attraction rather than repellency, an effect also noted in some previous coil evaluations. The increase in *Culex* captures in the coil arms can be explained by the known behavioral modes of action of pyrethroid-based coils and the genus-specific sensitivity of *Culex* to these compounds. Although coils are marketed as repellents, pyrethroid-based coils often function primarily as irritants or excito-repellents rather than true spatial repellents, leading to increased mosquito movement or displacement toward traps (2, 8, 18). *Culex* species in particular exhibit weaker spatial-repellency and knockdown responses to pyrethroid vapors compared with *Aedes* and *Anopheles*, which may explain the observed increase in captures (19, 20).

Dunnett’s multiple comparisons further clarified these patterns. In the urban environment, significant reductions relative to the control were observed for some products against *Aedes*, but only the Dynatrap^®^ and Thermacell^®^ devices produced significant reductions for *Culex*. No product at the urban site achieved statistical significance for *Anopheles*, likely due to low baseline abundance. At the rural site, nearly all products except the citronella candle and the ultrasonic device produced significant reductions in *Anopheles*, with the strongest effects again observed for the Dynatrap^®^ and Thermacell^®^ devices. For *Culex*, only these two devices significantly outperformed the control.

Taken together, these results show that the two tested heat-activated volatile pyrethroid devices are the only type of consumer-available spatial repellents that reliably provide strong, broad-spectrum protection across different environments and mosquito genera. Their performance was consistent in both high-density *Aedes* areas and rural landscapes dominated by *Anopheles*, supporting their use as dependable tools for decreasing human–mosquito contact. In contrast, citronella candles and ultrasonic devices should not be relied upon for meaningful protection, and although mosquito coils offer some benefit, they do not match the consistency or magnitude of protection provided by heat-activated volatile pyrethroid technologies.

Although this study was conducted during the dry season, when mosquito abundance in general was naturally lower, the objective was to compare device performance under consistent field conditions rather than to characterize peak-season transmission dynamics. The absence of *Aedes* at the rural site during the dry-season sampling period is consistent with long-standing patterns we routinely observe in this region, where *Aedes* populations decline sharply outside the rainy season. Nonetheless, evaluating these products during the wet-season period of highest mosquito pressure would be a valuable future direction to confirm whether the performance patterns observed here hold under peak epidemiological conditions.

An additional consideration is the role of the emanation mechanism itself. Heat-based devices volatilize active ingredients without combustion, whereas candles and coils rely on burning, a process that can degrade or combust the active ingredient before it disperses. This difference may partly explain the superior performance of heat-activated pyrethroid devices and the inconsistent performance of coils and candles. Essential-oil-based spatial repellents might similarly benefit from heat−based delivery systems that avoid combustion-related loss of active compounds, although this remains to be empirically tested.

Relatedly, pyrethroid-based emanators function through both odor-mediated spatial repellency and low-dose insecticidal or irritant effects. As noted earlier in this discussion, these mechanisms likely operate simultaneously, and their relative contributions may vary by species and environmental context. Continued reliance on pyrethroid-based spatial repellents raises the possibility that resistance could diminish both odor-mediated and toxic effects. This highlights the need to identify and evaluate new classes of spatial repellents with distinct modes of action to maintain long-term effectiveness.

Finally, these trials used CO_2_-baited traps as standardized proxies for human hosts. Although this approach allows controlled comparisons across products, it does not fully replicate the complex suite of cues emitted by humans. Highly anthropophilic mosquito strains may exhibit stronger attraction to humans than to traps, potentially overcoming the protective effect of spatial repellents under real-world conditions. Future studies incorporating human landing catches or semi-field systems with human participants would be important for determining how well the observed repellency translates to actual human protection, however, it would negate the health benefit of using non-human testing systems such as traps.

As global interest in non-contact vector control tools continues to grow, particularly in peri-domestic and outdoor settings, the results shown here emphasize the importance of evidence-based product choices. These findings support the effectiveness of heat-activated pyrethroid technologies as practical components of integrated mosquito management strategies and highlight the need for standardized, wind-controlled field testing to accurately assess consumer repellent performance.

## Data Availability

The raw data supporting the conclusions of this article will be made available by the authors, without undue reservation.

## Supplementary material

Supplementary Table 1 Urban site ANOVA results and effect sizes (η²).

Supplementary Table 2 Rural site ANOVA results and effect sizes (η²).
