# Supplementary material for: Performance of different spatial repellents (spatial emanators) against vector mosquito species in Mali, West Africa: a field trial using a non-human test method
Source: Front Insect Sci. 2026 Apr 21;6:1811511. doi: 10.3389/finsc.2026.1811511 (PMC13140856; doi:10.3389/finsc.2026.1811511)
Supplement: Supplementary Table 1 — Urban site ANOVA results and effect sizes (η²). [file Table1.docx]

**Supplementary Table S1**. Urban site ANOVA results and effect sizes (η²).

| **Source of Variation** | **SS** | **DF** | **MS** | **F (DFn, DFd)** | **P value** | **η²** |
| --- | --- | --- | --- | --- | --- | --- |
| Species | 1199 | 2 | 599.6 | F(2, 147) = 64.48 | < 0.0001 | 0.250 |
| Product | 1620 | 6 | 270.1 | F(6, 147) = 29.04 | < 0.0001 | 0.338 |
| Species × Product | 602.1 | 12 | 50.18 | F(12, 147) = 5.396 | < 0.0001 | 0.126 |
| Residual | 1367 | 147 | 9.299 | — | — | 0.285 |
