# Supplementary material for: Performance of different spatial repellents (spatial emanators) against vector mosquito species in Mali, West Africa: a field trial using a non-human test method
Source: Front Insect Sci. 2026 Apr 21;6:1811511. doi: 10.3389/finsc.2026.1811511 (PMC13140856; doi:10.3389/finsc.2026.1811511)
Supplement: Supplementary Table 2 — Rural site ANOVA results and effect sizes (η²). [file Table2.docx]

**Supplementary Table S2.** Rural site ANOVA results and effect sizes (η²).

| **Source of Variation** | **SS** | **DF** | **MS** | **F (DFn, DFd)** | **P value** | **η²** |
| --- | --- | --- | --- | --- | --- | --- |
| Species | 9394 | 2 | 4697 | F(2, 147) = 66.86 | < 0.0001 | 0.301 |
| Product | 5349 | 6 | 891.5 | F(6, 147) = 12.69 | < 0.0001 | 0.172 |
| Species × Product | 6122 | 12 | 510.1 | F(12, 147) = 7.262 | < 0.0001 | 0.196 |
| Residual | 10327 | 147 | 70.25 | — | — | 0.331 |
